# Baseline Serum Albumin for Long-Term Risk Stratification in Maintenance Hemodialysis Patients: A Retrospective Cohort Study

**DOI:** 10.3390/jcm15010333

**Published:** 2026-01-01

**Authors:** Kürşad Öneç, Gülşah Altun, Tansu Sav

**Affiliations:** Department of Nephrology, Faculty of Medicine, Duzce University, Duzce 81620, Türkiye; gulsahaltuntas11@gmail.com (G.A.); savtansu@gmail.com (T.S.)

**Keywords:** hemodialysis, albumin, mortality, prognosis, inflammation, risk stratification

## Abstract

**Background/Objectives:** Mortality among patients receiving maintenance hemodialysis remains high, and biomarkers that allow early risk stratification are needed. Serum albumin reflects nutritional status and systemic inflammation and has been associated with adverse outcomes; however, its long-term prognostic significance remains incompletely defined. This study examined the association between baseline serum albumin and long-term (up to 10-year) all-cause mortality in a large hemodialysis cohort. **Methods:** This retrospective cohort study included adult patients undergoing maintenance hemodialysis between 2015 and 2025 at a tertiary nephrology center. Individuals with at least three months of stable dialysis and available baseline serum albumin measurements were included. Patients were categorized into two groups according to baseline serum albumin levels (<3.5 g/dL and ≥3.5 g/dL). The primary outcome was long-term (up to 10-year) all-cause mortality, while secondary outcomes included emergency department visits, hospital admissions, cardiovascular events, and infection-related hospitalizations. Survival was assessed using Kaplan–Meier analysis, and predictors of mortality were evaluated using Cox proportional hazards regression. The median follow-up duration was 54 months (interquartile range: 28–92), with a maximum follow-up of 10 years. **Results:** A total of 412 patients were analyzed, of whom 40.8% had serum albumin levels < 3.5 g/dL. During follow-up, 233 deaths occurred. Lower albumin levels were associated with significantly higher mortality (76.2% vs. 43.4%, *p* < 0.001), increased healthcare utilization, and a greater incidence of cardiovascular and infectious complications. In multivariate analysis, albumin < 3.5 g/dL remained an independent predictor of mortality (hazard ratio 1.84, 95% confidence interval 1.42–2.38; *p* < 0.001). Receiver operating characteristic analysis identified 3.4 g/dL as the optimal cutoff for mortality prediction (area under the curve 0.72). **Conclusions:** Baseline serum albumin is an independent predictor of long-term (up to 10-year) mortality and adverse clinical outcomes in patients receiving maintenance hemodialysis. Although albumin is not a causal determinant, its association with survival likely reflects underlying nutritional and inflammatory burden. Prospective multicenter studies are warranted to validate albumin-based risk stratification and to evaluate the prognostic value of longitudinal changes in serum albumin over time.

## 1. Introduction

End-stage kidney disease requiring maintenance hemodialysis remains a major global health burden, with affected patients experiencing markedly reduced survival compared with the general population. Despite advances in dialysis technology, vascular access care, and infection control, long-term mortality rates in this population remain unacceptably high [1,2,3]. Identifying simple, reliable, and widely accessible biomarkers that can predict survival is therefore a key priority in nephrology as such indicators may facilitate early risk stratification and guide more individualized patient management [4,5].

Serum albumin is one of the most extensively studied prognostic markers in chronic hemodialysis patients. Although traditionally regarded as a marker of nutritional status, albumin is now understood to reflect a complex interplay of inflammation, oxidative stress, hepatic synthetic function, and overall metabolic reserve [6,7]. Hypoalbuminemia can result from malnutrition, chronic inflammation, comorbid illness, and dialysis-related factors, making it a robust yet nonspecific indicator of global physiological stress [8]. Numerous studies have demonstrated that lower serum albumin levels are strongly associated with increased hospitalization, cardiovascular morbidity, infection-related complications, and all-cause mortality in the hemodialysis population [9,10]. A threshold of 3.5 g/dL is commonly used in clinical practice to differentiate patients at higher risk, as values below this level consistently correlate with poorer clinical outcomes [11,12].

However, many of the available studies have been limited by relatively short follow-up durations, modest sample sizes, or heterogeneous patient groups. Data examining the long-term prognostic significance of baseline albumin levels over extended periods, such as a decade or more, remain scarce [13,14,15,16]. Furthermore, long-term survival data from regional populations, including those in Türkiye, are limited despite potential geographic differences in comorbidity patterns, dialysis practices, nutritional status, and inflammatory burden. Therefore, evaluating the prognostic value of serum albumin in a large hemodialysis cohort with a up to 10-year follow-up may provide important insights relevant to both clinical decision-making and regional healthcare planning.

The objective of this study was to investigate the association between baseline serum albumin levels and up to 10-year all-cause mortality in adult patients receiving maintenance hemodialysis. In addition to mortality, we examined the relationship between albumin levels and key clinical outcomes, including emergency department utilization, hospital admissions, cardiovascular events, and infection-related hospitalizations. By analyzing a decade-long dataset from a single regional hemodialysis center, the study aims to clarify the independent prognostic value of serum albumin and identify clinically meaningful thresholds that may enhance risk prediction in everyday practice.

## 2. Materials and Methods

### 2.1. Study Design and Setting

This study was designed as a retrospective cohort analysis conducted at the Department of Nephrology, Düzce University Faculty of Medicine. The institutional hemodialysis program serves a large regional population, allowing comprehensive evaluation of long-term clinical outcomes. All data were obtained from the electronic hospital information system and the dialysis unit registry, which contain routinely collected clinical, laboratory, and follow-up records. The retrospective design enabled the inclusion of a complete up to 10-year observation period without altering routine clinical care. No patient was contacted or examined specifically for research purposes, and no diagnostic or therapeutic intervention was introduced as part of the study protocol.

### 2.2. Ethical Approval

The study was conducted in accordance with the ethical principles of the Declaration of Helsinki and relevant national regulations governing retrospective clinical research. Approval was granted by the Düzce University Non-Interventional Clinical Research Ethics Committee (Decision No: 2025/289; Date: 10 November 2025). Because the analysis was based entirely on anonymized retrospective data, the requirement for informed consent was waived by the ethics committee.

### 2.3. Study Population

The study population consisted of adult patients (≥18 years) who received maintenance hemodialysis at Düzce University between January 2015 and January 2025. Eligible patients were required to have been on hemodialysis for at least three consecutive months to ensure clinical stability and adequate dialysis exposure. Additionally, availability of a baseline serum albumin measurement and complete mortality information for a follow-up period of up to 10 years was required. Patients undergoing dialysis due to acute kidney injury, those with a history of kidney transplantation, individuals who transitioned to peritoneal dialysis, and those with terminal non-renal diseases or active malignancies at baseline were excluded. Records with missing essential laboratory or clinical variables were also omitted to maintain the accuracy and consistency of data analyses. Due to the retrospective nature of the study, transient conditions such as acute infection or gastrointestinal disturbances (e.g., diarrhea) at the time of baseline albumin measurement could not be systematically identified and were therefore not included as formal exclusion criteria.

### 2.4. Data Collection

Data were extracted from the electronic medical record system, which archives routine laboratory values, comorbidities, dialysis session information, hospitalization data, and longitudinal follow-up notes. Demographic variables included age at baseline and sex. Clinical variables encompassed duration of dialysis expressed in months, presence of diabetes mellitus, hypertension, and established cardiovascular disease, as well as vascular access type. Baseline laboratory parameters were defined as the first available measurement closest to the index date and included serum albumin, hemoglobin, creatinine, phosphorus, calcium, sodium, potassium, and C-reactive protein.

All laboratory analyses were performed in the same institutional biochemistry laboratory using standardized automated analyzers. Serum albumin, creatinine, electrolytes, calcium, and phosphorus measurements were conducted using an AU5800 Clinical Chemistry Analyzer (Beckman Coulter Inc., Brea, CA, USA). Hemoglobin levels were measured using an automated hematology analyzer (XN-1000; Sysmex Corporation, Kobe, Japan). C-reactive protein levels were determined by immunoturbidimetric assay using the same chemistry analyzer platform. Routine internal quality control procedures were applied daily, and external quality assurance programs were conducted regularly throughout the study period to ensure analytical accuracy and consistency of measurements.

### 2.5. Exposure Definition

The primary exposure variable was baseline serum albumin level. Albumin was analyzed both as a continuous variable and as a dichotomous categorical variable using the widely accepted clinical threshold of 3.5 g/dL. Patients with serum albumin levels below 3.5 g/dL were classified as having hypoalbuminemia, while those with albumin levels of 3.5 g/dL or higher were considered to have preserved nutritional and inflammatory status. This cutoff has been consistently used in the nephrology literature to distinguish patients at elevated risk of poor outcomes.

### 2.6. Outcome Measures

The primary outcome of the study was up to 10-year all-cause mortality. Mortality status and dates were verified through hospital records and the national death notification system. Secondary outcomes included annual frequency of emergency department visits, number of hospital admissions per year, occurrence of cardiovascular events during follow-up, and infection-related hospitalizations. These outcomes were chosen to capture both the long-term survival and the clinical burden associated with varying albumin levels in hemodialysis patients.

### 2.7. Follow-Up

Follow-up duration for each patient was calculated from the date of baseline albumin measurement to the date of death or the end of the observation period, whichever occurred first. Patients who were alive at the end of follow-up were censored at the study closure date. No patient was lost to follow-up with regard to mortality status, as the national reporting system systematically documents all deaths occurring within the country. Patients were followed until death or 31 January 2025 (study end).

### 2.8. Statistical Analysis

All statistical analyses were performed using IBM SPSS Statistics version 26.0 (IBM Corp., Armonk, NY, USA). Continuous variables were evaluated for normality using the Kolmogorov–Smirnov test and visual assessment of distribution plots. Normally distributed variables were summarized as mean ± standard deviation, while non-normally distributed variables were expressed as median and interquartile range. Categorical variables were presented as frequencies and percentages.

Between-group comparisons were conducted according to the distributional properties of each variable. Student’s *t*-test was used for normally distributed continuous variables, and the Mann–Whitney U test was applied for non-normally distributed parameters. Categorical variables were compared using the chi-square test.

Survival analyses were performed using the Kaplan–Meier method, and differences in survival between albumin categories were assessed with the log-rank test.

To identify factors associated with up to 10-year all-cause mortality, univariate Cox proportional hazards regression analyses were first performed for demographic, clinical, and laboratory variables, including age, sex, dialysis duration, comorbidities, hemoglobin, C-reactive protein, phosphorus, and serum albumin (evaluated both as a continuous and categorical variable). Variables with a *p*-value < 0.10 in univariate analyses were entered into a multivariate Cox regression model to determine independent predictors of mortality. Hazard ratios and 95% confidence intervals were reported.

The predictive performance of baseline serum albumin levels for long-term mortality was further assessed using receiver operating characteristic (ROC) curve analysis. The area under the curve was calculated, and the optimal cutoff value was identified based on the Youden index, along with sensitivity, specificity, positive predictive value, and negative predictive value. A two-sided *p*-value < 0.05 was considered statistically significant for all analyses.

## 3. Results

A total of 412 hemodialysis patients met the inclusion criteria and were analyzed. Among them, 168 patients (40.8%) had serum albumin levels < 3.5 g/dL, whereas 244 patients (59.2%) had albumin levels ≥ 3.5 g/dL. The median follow-up duration was 54 months (interquartile range: 28–92), with a maximum follow-up of 10 years.

As shown in Table 1, patients in the low-albumin group were significantly older (68.2 ± 12.8 vs. 60.1 ± 12.4 years, *p* < 0.001) and had a longer dialysis duration (61 vs. 46 months, *p* = 0.004). The prevalence of diabetes (60.7% vs. 40.6%, *p* < 0.001) and cardiovascular disease (40.5% vs. 26.2%, *p* = 0.003) was also higher among those with hypalbuminemia. Laboratory results demonstrated significantly lower hemoglobin (9.9 ± 1.6 vs. 10.7 ± 1.4 g/dL, *p* < 0.001) and higher CRP (15.8 vs. 8.7 mg/L, *p* < 0.001) in the low-albumin group.

During the follow-up period of up to 10 years, 233 deaths (56.6%) occurred. As summarized in Table 2, mortality was substantially higher in patients with serum albumin < 3.5 g/dL compared with those with preserved levels (76.2% vs. 43.4%, *p* < 0.001), corresponding to a significantly increased risk of death (HR 1.84, 95% CI 1.42–2.38; *p* < 0.001). Low-albumin patients also demonstrated more frequent emergency department visits (median 3 vs. 2 per year, *p* = 0.002) and hospital admissions (2 vs. 1 per year, *p* = 0.008). Cardiovascular events (35.1% vs. 19.7%, *p* = 0.001) and infection-related hospitalizations (42.3% vs. 25.4%, *p* < 0.001) were significantly more common in the low-albumin group.

Kaplan–Meier survival curves demonstrated a clear and progressive separation between the two albumin groups throughout the up to 10-year follow-up period (log-rank *p* < 0.001). As summarized in Table 3, patients with albumin < 3.5 g/dL showed substantially lower survival probabilities at every time point, beginning as early as the first year (86.3% vs. 95.1%) and widening steadily over time. By year 10, survival had declined to 23.8% in the low-albumin group compared with 56.6% among patients with albumin ≥ 3.5 g/dL, highlighting the long-term prognostic significance of baseline hypoalbuminemia.

Univariate Cox regression results are summarized in Table 4. Several variables showed significant associations with up to 10-year all-cause mortality. Age demonstrated a consistent incremental risk (HR 1.04 per year, *p* < 0.001), while diabetes and longer dialysis duration were also associated with higher mortality. Laboratory parameters reflecting nutritional and inflammatory status were strong predictors, including lower hemoglobin (HR 0.87, *p* < 0.001) and elevated CRP (HR 1.02, *p* < 0.001). Serum albumin was analyzed both as a continuous variable and as a categorical variable using the 3.5 g/dL threshold.

Albumin showed the strongest effect among laboratory markers, both as a continuous measure (HR 1.18 per 0.1 g/dL decrease, *p* < 0.001) and as a categorical variable (<3.5 g/dL; HR 2.11, *p* < 0.001), indicating a substantial increase in risk among patients with hypoalbuminemia.

In the multivariate Cox regression model (Table 5), serum albumin < 3.5 g/dL remained an independent predictor of up to 10-year all-cause mortality after adjustment for demographic, clinical, and inflammatory variables (HR 1.84, 95% CI 1.42–2.38; *p* < 0.001). Age demonstrated a consistent and clinically meaningful association with mortality (HR 1.03 per year, *p* < 0.001), while diabetes also retained independent significance (HR 1.41, *p* = 0.005). CRP showed a modest but statistically significant association (HR 1.01, *p* = 0.032), supporting the contribution of systemic inflammation to long-term risk. Hemoglobin, although lower in non-survivors, did not reach statistical significance in the adjusted model (*p* = 0.068), suggesting that its effect may be mediated through other comorbid or inflammatory pathways rather than acting as a standalone predictor.

As shown in Table 6, serum albumin demonstrated acceptable discriminatory ability for predicting up to 10-year all-cause mortality, with an AUC of 0.72. The optimal cutoff value identified by the Youden index was 3.4 g/dL, which provided a sensitivity of 71.8% and a specificity of 65.2%, indicating a balanced performance in detecting high-risk patients. The corresponding positive and negative predictive values (68.4% and 69.0%, respectively) further suggest that albumin may serve as a practical and clinically meaningful marker for long-term risk stratification in the hemodialysis population.

## 4. Discussion

In this retrospective cohort study of patients undergoing maintenance hemodialysis, lower baseline serum albumin levels were strongly associated with increased all-cause mortality at a follow-up period of up to 10 years and a higher burden of adverse clinical outcomes. Although albumin has long been regarded as a nutritional biomarker, our findings reinforce its broader role as an integrated indicator of inflammation, metabolic reserve, and overall physiologic resilience in the hemodialysis population [17,18]. The extended follow-up period and sizable cohort allowed for a more comprehensive evaluation of the prognostic significance of albumin compared with previous reports with shorter observation windows.

The association between hypoalbuminemia and mortality is likely mediated by multiple overlapping mechanisms. Inflammation is a central component of this relationship, as proinflammatory cytokines suppress hepatic albumin synthesis, increase vascular permeability, and promote protein catabolism [19,20]. Consistent with these mechanisms, patients with lower albumin levels in our cohort also demonstrated substantially higher CRP levels, suggesting that a chronic inflammatory milieu may play a major contributing role. Albumin’s antioxidant properties and its capacity to bind circulating toxins provide additional physiological pathways linking lower concentrations to increased vulnerability to cardiovascular and infectious complications [21].

The markedly higher mortality at up to 10 years observed in the low-albumin group may therefore reflect a combination of malnutrition, persistent inflammation, and impaired host defense. Chronic inflammation and malnutrition are known to predispose patients to both infection-related morbidity and cardiovascular instability, and this pattern was evident in our findings, with higher rates of cardiovascular events and infection-related hospitalizations in the hypoalbuminemic group. These clinical associations align with the concept that albumin reflects global physiologic robustness rather than a single isolated biological pathway [22,23].

Health service utilization patterns in our study further underscore the clinical relevance of albumin. Patients with lower albumin levels had significantly more emergency department visits and hospital admissions, indicating greater clinical instability and a higher burden of acute events. This observation supports the notion that albumin may function not only as a mortality predictor but also as a marker of overall health system burden in the hemodialysis population [14,24].

Other clinical variables demonstrated expected associations with mortality. Age remained an independent predictor, likely due to the cumulative impact of vascular stiffness, immunosenescence, comorbidity accumulation, and diminished regenerative capacity in older individuals [25]. Diabetes was also independently associated with mortality, a finding consistent with its established role in predisposing patients to both macrovascular and microvascular complications, chronic inflammation, and impaired immune responses. The independent effect of CRP reinforces the central importance of inflammation in hemodialysis-related morbidity and mortality and highlights the interplay between inflammatory burden and albumin metabolism.

Although hemoglobin levels were lower in patients with hypoalbuminemia, hemoglobin did not retain independent statistical significance in the multivariate model. This finding may reflect the multifactorial nature of anemia in hemodialysis, which is influenced by erythropoietin responsiveness, iron availability, chronic inflammation, and dialysis adequacy. The absence of an independent survival effect may indicate that anemia contributes less to long-term mortality than broader inflammatory and nutritional factors [26,27].

Phosphorus levels were not independently associated with mortality in this cohort. While hyperphosphatemia has been linked to vascular calcification and mortality, phosphorus concentrations may have been relatively well controlled in our center, reducing the variability needed to detect an independent effect. Alternatively, phosphorus-related risk may have been overshadowed by stronger prognostic markers such as albumin and inflammatory status [28,29].

The ROC analysis revealed that an albumin threshold of 3.4 g/dL provided the best balance of sensitivity and specificity for predicting long-term mortality. Although this cutoff aligns with commonly used clinical thresholds, caution is warranted, as optimal values may vary depending on population characteristics, nutritional profiles, and inflammation prevalence. Validation in independent cohorts is therefore necessary before firm recommendations can be made.

Validation in independent cohorts is therefore necessary before firm recommendations can be made. In addition to individual cohort studies, evidence from systematic reviews and meta-analyses further supports the robust association between serum albumin levels and mortality in dialysis populations [30,31,32,33]. Pooled analyses encompassing diverse hemodialysis cohorts have consistently demonstrated that lower serum albumin concentrations are associated with a significantly increased risk of all-cause mortality, independent of age, comorbidity burden, and dialysis vintage. These findings suggest that the prognostic value of albumin is preserved across different geographic regions and clinical settings, reinforcing its role as a reliable marker of long-term risk stratification rather than a population-specific observation

Recent evidence also supports the prognostic importance of dynamic albumin measurements rather than static baseline values alone. For example, a recent multicohort study using robust joint models demonstrated that serial serum albumin trajectories significantly improved mortality predictions in dialysis patients compared with conventional Cox models, with consistent hazard ratios observed across models ranging from 1.22 to 1.28 for death across both peritoneal and hemodialysis cohorts. These findings suggest that incorporating time-dependent patterns of albumin change may further enhance risk stratification beyond baseline albumin levels alone and underscore the potential value of longitudinal biomarker monitoring in clinical practice [34]. Our findings, focusing on baseline albumin, align with this emerging evidence and suggest that future studies incorporating longitudinal albumin changes could yield even more accurate prognostic models.

This study has several limitations that should be acknowledged. First, the retrospective design precludes causal inference, and the associations identified may be influenced by unmeasured confounders. Albumin levels were assessed only at baseline, and temporal changes in albumin—which may better reflect evolving nutritional or inflammatory states—were not evaluated. Detailed assessments of dietary intake, gastrointestinal losses, hepatic function, or hydration status were unavailable and may have contributed to albumin variability. Similarly, inflammatory markers other than CRP, such as interleukin-6, ferritin, or TNF-α, were not measured and could have provided a more nuanced understanding of the inflammatory milieu. The study was conducted at a single center, which may limit generalizability to other geographic regions or dialysis populations with differing demographic or clinical characteristics. Finally, cause-specific mortality data were not uniformly accessible, necessitating the use of all-cause mortality as the primary endpoint. This limitation may obscure potential differences in cardiovascular versus infection-related death patterns.

In addition, transient clinical conditions such as acute infection or gastrointestinal disturbances at the time of baseline albumin measurement could not be systematically captured due to the retrospective design and may have contributed to short-term variability in albumin levels.

## 5. Conclusions

In conclusion, baseline serum albumin levels were strongly associated with up to 10-year all-cause mortality and multiple adverse clinical outcomes in patients undergoing maintenance hemodialysis. Although albumin cannot be considered a causal factor, its association with mortality likely reflects a composite of malnutrition, inflammation, impaired metabolic reserve, and increased susceptibility to both cardiovascular and infectious complications. These findings suggest that albumin may serve as a practical and readily accessible tool to support long-term risk stratification in the hemodialysis population. However, given the observational design and the potential influence of unmeasured confounding factors, further prospective and multi-center studies are needed to validate the clinical utility of albumin-based risk classification and to explore whether dynamic changes in albumin over time offer additional prognostic value.

## Figures and Tables

**Table 1:** Baseline Characteristics of the Study Population. Baseline demographic, clinical, and laboratory characteristics of the study population, stratified according to baseline serum albumin levels (<3.5 g/dL vs. ≥3.5 g/dL).

Variable	Overall (n = 412)	Albumin < 3.5 g/dL (n = 168)	Albumin ≥ 3.5 g/dL (n = 244)	*p*-Value
Age, years	63.4 ± 13.1	68.2 ± 12.8	60.1 ± 12.4	<0.001
Female, n (%)	189 (45.9%)	83 (49.4%)	106 (43.4%)	0.234
Dialysis duration, months	52 (18–94)	61 (24–110)	46 (16–80)	0.004
Diabetes, n (%)	201 (48.8%)	102 (60.7%)	99 (40.6%)	<0.001
Hypertension, n (%)	316 (76.7%)	133 (79.1%)	183 (75.0%)	0.357
Cardiovascular disease, n (%)	132 (32.0%)	68 (40.5%)	64 (26.2%)	0.003
Albumin, g/dL	3.55 ± 0.47	3.17 ± 0.24	3.79 ± 0.32	<0.001
Hemoglobin, g/dL	10.4 ± 1.5	9.9 ± 1.6	10.7 ± 1.4	<0.001
Creatinine, mg/dL	7.63 ± 2.14	7.51 ± 2.41	7.71 ± 1.94	0.402
CRP, mg/L	11.4 (5.6–21.9)	15.8 (8.1–28.4)	8.7 (4.1–17.2)	<0.001
Phosphorus, mg/dL	4.9 ± 1.3	4.8 ± 1.4	5.0 ± 1.2	0.186
Sodium, mmol/L	137.8 ± 3.4	137.4 ± 3.6	138.1 ± 3.2	0.078
Potassium, mmol/L	4.9 ± 0.7	4.8 ± 0.7	4.9 ± 0.7	0.221

CRP: C-reactive protein.

**Table 2:** Clinical Outcomes According to Albumin Categories. Comparison of mortality and secondary clinical outcomes, including healthcare utilization and complication rates, between patients with low and preserved baseline serum albumin levels during follow-up.

Outcome	Albumin < 3.5 (n = 168)	Albumin ≥ 3.5 (n = 244)	*p*-Value
up to 10-year mortality, n (%)	128 (76.2%)	105 (43.4%)	<0.001
Annual ED visits	3 (1–6)	2 (0–4)	0.002
Annual hospital admissions	2 (1–4)	1 (0–3)	0.008
Cardiovascular events, n (%)	59 (35.1%)	48 (19.7%)	0.001
Infection-related hospitalization, n (%)	71 (42.3%)	62 (25.4%)	<0.001

ED: emergency department.

**Table 3:** Kaplan–Meier Survival Estimates. Kaplan–Meier survival probabilities at predefined time points for patients stratified by baseline serum albumin levels, with comparisons assessed using the log-rank test.

Time Point	Albumin < 3.5 (%)	Albumin ≥ 3.5 (%)	Log-Rank *p*-Value
1-year	86.3	95.1	<0.001
3-year	67.4	83.5	
5-year	52.8	72.2	
up to 10-year	23.8	56.6	

**Table 4:** Univariate Cox Regression Analysis. Univariate Cox proportional hazards regression analysis evaluating demographic, clinical, and laboratory variables associated with up to 10-year all-cause mortality.

Variable	HR	95% CI	*p*-Value
Age	1.04	1.03–1.05	<0.001
Female sex	1.12	0.88–1.42	0.344
Diabetes	1.51	1.21–1.89	<0.001
Dialysis duration	1.01	1.00–1.01	0.014
Albumin (per 0.1 g/dL decrease)	1.18	1.12–1.24	<0.001
Albumin < 3.5 g/dL	2.11	1.69–2.64	<0.001
Hemoglobin	0.87	0.80–0.94	<0.001
CRP	1.02	1.01–1.03	<0.001
Phosphorus	1.08	0.97–1.21	0.163

HR: hazard ratio; CI: confidence interval; CRP: C-reactive protein.

**Table 5:** Multivariate Cox Regression Analysis. Multivariate Cox proportional hazards regression model identifying independent predictors of up to 10-year all-cause mortality after adjustment for relevant demographic, clinical, and inflammatory variables.

Variable	Adjusted HR	95% CI	*p*-Value
Age	1.03	1.02–1.04	<0.001
Diabetes	1.41	1.11–1.80	0.005
Dialysis duration	1.00	1.00–1.01	0.091
Albumin < 3.5 g/dL	1.84	1.42–2.38	<0.001
Hemoglobin	0.93	0.85–1.01	0.068
CRP	1.01	1.00–1.02	0.032

CRP: C-reactive protein. HR: hazard ratio; CI: confidence interval;

**Table 6:** ROC Analysis of Albumin Predicting Mortality. Receiver operating characteristic (ROC) curve analysis evaluating the discriminatory performance of baseline serum albumin for predicting up to 10-year all-cause mortality.

Parameter	Value
AUC	0.72
Optimal cutoff	3.4 g/dL
Sensitivity	71.8%
Specificity	65.2%
PPV	68.4%
NPV	69.0%

AUC: area under the curve; PPV: positive predictive value; NPV: negative predictive value.

## Data Availability

The data presented in this study are available upon reasonable request from the corresponding author. The data are not publicly available due to ethical and privacy restrictions.

## Abbreviations

AUC	Area Under the Curve
CI	Confidence Interval
CKD	Chronic Kidney Disease
CRP	C-Reactive Protein
ED	Emergency Department
ESRD	End-Stage Renal Disease
HD	Hemodialysis
HR	Hazard Ratio
IQR	Interquartile Range
KM	Kaplan–Meier
NPV	Negative Predictive Value
PPV	Positive Predictive Value
ROC	Receiver Operating Characteristic
